# Choice of alcohol over a natural reward: an experimental study in light and heavy social drinkers

**DOI:** 10.1007/s00213-024-06679-6

**Published:** 2024-10-03

**Authors:** Hanna Karlsson, Sarah Mcntyre, Sarah Gustavson, David Andersson, Ilona Szczot, Markus Heilig, Irene Perini

**Affiliations:** 1 Center for Social and Affective Neuroscience, Department of Biomedical and Clinical Sciences, Linköping University, Psychiatry Building, 58183 Linköping, Sweden; 2 Department of Management and Engineering, Division of Economics, Linköping University, 58183 Linköping, Sweden; 3 Center for Medical Image Science and Visualization (CMIV), Linköping University, 58183 Linköping, Sweden

**Keywords:** Alcohol, Substance use disorder, Heavy drinking, Addiction, Choice, Reward

## Abstract

**Rationale & Objectives:**

A core symptom of alcohol use disorder (AUD) is a progressively increased choice of alcohol over alternative rewards despite negative consequences. Here, we investigated choice between personalized alcohol vs. natural rewards in a laboratory setting, and compared this behavior between non-treatment-seeking heavy drinkers and light social drinkers.

**Methods:**

30 light social drinkers (15 men drinking < 15 drinks/week and 15 women drinking < 10 drinks/week) and 30 heavy, non-treatment-seeking drinkers (drinking more than these levels; 15 women). In the Concurrent Choice Alcohol Food (CCAF) task, participants chose between individually tailored images of alcohol and snack rewards and collected points towards the respective reward. To assess cost sensitivity, points associated to the images varied so that they favored alcohol or snack, or were equal, creating three relative point levels.

**Results:**

Choice preference for alcohol was strongly correlated with Alcohol Use Disorder Identification Test (AUDIT) scores, supporting the external validity of the choice procedure. Compared to light drinkers, heavy drinkers showed increased choice preference for alcohol, as indicated by a between-group difference in points of subjective equality, a metric that quantifies the relative point level at which alcohol and snacks were equally likely to be chosen. In both groups, choice preference strongly depended on the relative point level of alcohol compared to snacks, suggesting that responding for alcohol in heavy drinkers was sensitive to costs.

**Conclusions:**

Our results replicate previous findings of a relationship between self-reported alcohol use and choice preference for alcohol. We also found that choice behavior was strongly dependent on relative cost of alcohol in both groups, although price sensitivity was lower in heavy compared to light drinkers. An increased choice preference for alcohol in heavy drinkers suggests that they attribute a higher relative reinforcing value to alcohol compared to natural rewards.

## Introduction

Alcohol use accounts for ~ 6% of global disease burden in men, and 1.6% in women (Griswold et al. 2018). Social drinking is widely accepted in Western societies and remains controlled in the majority of users. However, in a significant minority, difficulties in regulating use emerge over time, and alcohol use disorder (AUD) ultimately develops (Grant et al. 2015). This risk is elevated in people that engage in heavy drinking (Nieto et al. 2021). Commonly, AUD goes together with psychiatric and medical comorbidities, as well as interpersonal and financial problems (Dethier & Blairy 2012; Fink et al. 2016; Rehm et al. 2003), ultimately contributing to a vicious circle of alcohol-related problems that themselves promote relapse to heavy drinking (Sinha 2007). Despite this, patients with AUD continue to consume alcohol (Wagner & Anthony 2002), and increasingly choose alcohol over healthy rewards (American Psychiatric Association 2013; Heilig et al. 2019).

Factors that influence drug seeking and taking have commonly been studied in the absence of other rewards being available (Ahmed 2010). However, similar to what others have reported for opioids and stimulants (Ahmed et al. 2013; Banks & Negus 2017; Lenoir et al. 2007; Spragg 1936), recent research in animal models from our group has shown that availability of an alternative high-value reward is an important determinant of alcohol-related behaviors. When rats were presented with a mutually exclusive choice of alcohol and a sweet solution, a majority chose the sweet solution, but ~ 15% continued to choose alcohol, a percentage similar to human AUD rates. Decreased expression of the γ-aminobutyric acid (GABA) transporter GAT-3 in Central Amygdala (CeA) was identified a causal factor behind alcohol choice in these rats. Parallel evidence from human postmortem brain tissue showed similarly low GAT-3 expression in CeA of people with AUD, suggesting a translational validity of the rat findings (Augier et al. 2018).

Based on these choice models in experimental animals, mutually exclusive, concurrent choice between alcohol and an alternative reward has been used to provide insights into behavioral mechanisms in people (Hogarth & Hardy 2018). In a computer-based task, participants made a series of choices between alcohol and a snack by clicking on an image of the preferred reward. The study assessed whether choice preference for alcohol was associated with the individual level of alcohol use, and by costs imposed on alcohol-related choices. Costs were manipulated on a trial-by-trial level in two ways: by setting the relative point level to be in favor of a snack, and by introducing a delay imposed on the receipt of the reward following alcohol choice. Drinking severity was associated with assigning greater value to alcohol but not with sensitivity to costs associated with alcohol-related stimuli. These findings suggested that excessive alcohol choice in individuals with AUD might be driven by value-based choice rather than insensitivity to cost (Hogarth 2020). The association between drug use severity and preference for drug over an alternative reward has been established in several studies and across different drug categories, demonstrating replicability and generalizability of the finding. In addition, preference for drug using these concurrent choice tasks, was shown to be as sensitive as craving and consumption ratings to manipulations of abstinence, satiety, negative affect and drug cue exposure (Hogarth & Field 2020).

The aim of our study was to investigate whether heavy compared to light drinkers showed altered choice preference for alcohol over a concurrently presented natural reward. We hypothesized increased choice preference for alcohol in heavy drinkers compared to light drinkers as well as a maintained sensitivity for the cost of choosing alcohol, as previously shown by Hogarth et al. Here, we adapted the published alcohol choice task (Hogarth & Hardy 2018), and optimized it for future functional magnetic resonance imaging (fMRI) studies. We developed a simplified and personalized version of the original paradigm to assess choice preference for alcohol in non-treatment seeking heavy drinkers, and compared it with that of light social drinkers. To simplify the paradigm, costs were only assessed by three relative point levels instead of the five used originally. To increase the effect size of the task, alcohol and snack images were tailored to participants’ preference, assessed before the experimental session. Most studies using concurrent choice paradigms use predefined reward-related stimuli, which can introduce variance in the results due to between-subject variability in subjective preference, for example, when using branded items as reward-related stimuli. To reduce task noise, we used images that were tailored to participants’ preference, and that did not depict a specific brand. We examined the external validity of the task by assessing severity of alcohol problems using a validated, WHO-developed assessment instrument, the Alcohol Use Disorder Identification Test (AUDIT) (Saunders et al. 1993). Choice behavior was modelled using logistic regression, and from this we interpolated a novel metric, “point of subjective equality” (PSE), which gives the relative point level at which alcohol and snacks were equally likely to be chosen by an individual or group. In addition, comparing the slope of the logistic regression function between groups allows the investigation of a potential difference in cost sensitivity. The PSE metric may have utility as a biomarker of efficacy in future medication development programs as shown in other choice task in animals and humans previously (Hogarth & Chase 2012; Hogarth et al. 2015; Lile et al. 2016).

## Material and methods

### Participants

Healthy volunteers were recruited using advertisements on social media. Of 80 participants who were evaluated, 15 cancelled the session, 2 did not meet inclusion criteria and 3 were excluded prior to analysis because their age exceeded two STD from the mean. Participants were grouped into light social drinkers (LD, N = 30, 15 females) and non-treatment seeking heavy drinkers (HD, N = 30, 15 females) based on self-reported number of drinks per week. Heavy drinking was defined according to the Swedish public health agency, as 15 or more drinks per week for males and 10 or more drinks/week for women (Socialstyrelsen 2011).

Inclusion criteria included consumption of alcohol on one or more occasions in the past 3 months, age 20–65 years, minimum high school education and sufficient knowledge of Swedish to understand participant information and experimental instructions.

Subjects were excluded if they were currently receiving or seeking treatment for alcohol problems, had used illicit substances in the past month, had any current clinically significant psychiatric disorder, any past or present psychotic or bipolar disorder, had ongoing psychoactive medication, were pregnant or nursing, or had any history of clinically significant neurological disorders. To determine eligibility, the Modified MINI Screen (MMS) (Alexander et al. 2007) was used to screen for psychiatric disorders, and the Drug Use Disorder Identification Test (DUDIT) (Berman et al. 2007) and urine drug screening was used to identify illicit drug use. All women performed a urine pregnancy screening.

Baseline personality traits were obtained using the NEO Five Factor Inventory (NEO-FFI) (McCrae & Costa 1987). The Barratt Impulsiveness Scale (BIS) was used to assess the behavioral construct of impulsiveness (Barratt 1965). The Family Tree Questionnaire (FTQ) (Mann et al. 1985) was used to asses family history of AUD. AUDIT was used to assess alcohol use problems, and also served as proxy for the presence of AUD. We didn’t evaluate participants for a formal diagnosis of AUD, and a potential presence of AUD was not exclusionary. Given the AUDIT scores of participants in our heavy drinker group, and published data on the correlation between AUDIT scores and AUD diagnosis, all our participants in the heavy drinker group likely met criteria for moderate and severe AUD (Källmén et al. 2019; Moehring et al. 2019). However, participants were included only if they were not currently seeking or receiving treatment for alcohol problems; participants with clinically significant alcohol use problems were counselled after completion of the study, and offered referral to treatment.

The study was approved by the Swedish Ethical Review Authority (ref. 2020/07255) and all participants provided written informed consent.

### Study timeline

**Fig. 1:**
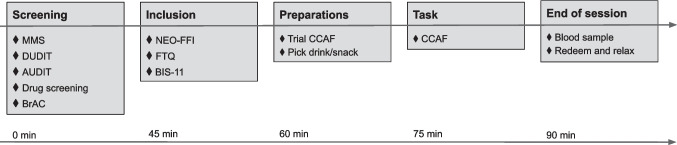
Study timeline. During screening *MMS* Modified Mini Screen, *DUDIT* Drug Use Disorders Identification Test, *AUDIT* Alcohol Use Disorder Identification Test were used and *BrAC* Breath Alcohol Concentration measured. After inclusion, participants filled out the *NEO-FFI* NEO Five-Factor Inventory, *FTQ* Family Tree Questionnaire and *BIS-11* Barratt Impulsiveness Scale. Participants received instructions and did a trial version of the Choice Alcohol-Food (CCAF) task. The task was then performed in front of a computer. After task completion, a blood sample was taken and finally, participants received the reward depending on CCAF task results

The study consisted of one afternoon visit, which included a screening assessment followed by a laboratory session (Fig. 1). Participants were asked to abstain from alcohol (72 h) and food (3 h) prior to the session.

During screening, prospective participants were evaluated for eligibility by a research nurse or a physician and, upon inclusion, filled out questionnaires. Participants were then invited to carry out two different tasks, assessing choice preference for alcohol and social processing (the latter not presented here).

Before carrying out the task, participants were presented with four alcoholic drinks (lager beer, vodka, red wine and white wine) and bowls containing four snacks (chocolate, chips, nuts, and candy), and asked to pick one alcoholic drink and one snack depending on their preference. At this stage, the subjects could hold and smell the glasses and bowls containing the rewards, but not consume them. Pictures of the chosen items were later used to tailor the stimuli to participants’ preferences. Task instructions were given, and the subjects then practiced the task in preparation for the experimental session.

After completing the experimental session, participants provided a blood sample for future genetic analyses. At the end of the session, all subjects received one standard drink of their preferred alcoholic drink or one serving of their favorite snack, depending on which of these reward categories they had earned most points for during the task. Pictures, task instructions and analysis scripts are available on GitHub: https://github.com/NeuroIP/CCAF.

### Concurrent choice alcohol-food (CCAF) task

**Fig. 2:**
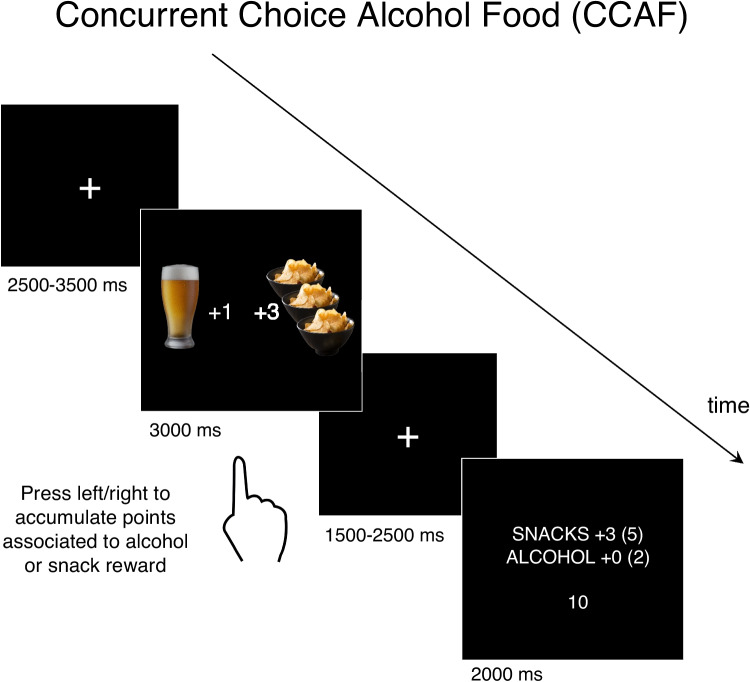
An illustrative Concurrent Choice Alcohol-Food (CCAF) task trial. Participants made forced choices between accumulating alcohol or food points. The accumulated points during the trial were then presented after a jittered fixation period. Total points were also displayed in brackets, and a trial countdown was presented at the bottom of the screen. Conditions were balanced based on relative point level (-2, + 0, + 2). The trial depicted here illustrates an increased relative point level for food compared to alcohol (-2 relative point level). Pictures associated with alcohol/snacks were based on the selections made by participants prior to the session

The CCAF task was modified from (Hogarth & Hardy 2018). Participants were instructed to collect points toward either an alcohol drink or a snack that they redeemed at the end of the session. They were told that the more points they collected toward a reward, the better the chance to receive that reward in the end. The CCAF task trial is presented in Fig. 2. During each trial, participants were first presented with two concurrent pictures showing their preferred snack and their preferred alcoholic drink (3000 ms). During this interval, participants chose one of the two pictures by clicking mouse buttons with their index or middle finger. Following their choice, there was a jittered fixation interval (1500–3500 ms), after which participants were presented with a feedback image (2000 ms), showing the points earned during the trial for the respective reward category, and the running total in brackets. Each alcohol and snack picture was associated with either 1 or 3 points, shown on the side of the picture. This feature introduced three relative point levels. When both pictures were associated with either 1 or 3 points, the relative point levels were equal (0). When the relative point level differed, it could be in favor of alcohol (+ 2) or snacks (-2). Each relative point level was balanced across trials, with 32 trials per relative level and 96 trials in total. The position of alcohol and snack images (left or right) was counterbalanced across trials. At the end of the session, participants who accumulated more alcohol points than snack points in total received one standard drink of their preferred alcoholic drink, while those who accumulated more snack points received a portion of their favorite snack.

### Statistical analysis

Sample size was based on a power analysis using G*Power, based on a within-subject factor with 3 levels (i.e. relative point level: -2, 0, + 2) and a between-subject factor with 2 levels (i.e. group: light drinkers, heavy drinkers). Assuming an effect size of Cohen’s f ≥ 0.31 (moderate effect size, as seen previously (Hogarth & Hardy 2018) and an α = 0.05, a total sample size of 58 subjects was required to detect a between-group behavioral effect with ≥ 80% power.

The individual percentage of alcohol choice, reflecting the number of trials when alcohol was chosen as a percentage of the total number of choice trials was calculated. Data were highly skewed, with near-ceiling and -floor effects for relative point levels + 2 and -2 respectively. Therefore, percentage of alcohol choice data were first logit transformed and then used for all subsequent analyses.

To identify predictors of percentage of alcohol choice, we entered relative point level (-2, 0, + 2) and group (light drinkers, heavy drinkers) as independent variables and sex (female, male) as covariate in a linear mixed-effects (lme) model.

As a metric of choice behavior, we assessed the relative point level at which alcohol and snacks were equally chosen in the two groups. First, percentage of alcohol choice data according to relative point level were separately fitted, using logistic regression, for heavy and light drinkers, respectively. Parametric bootstrapping was performed with 1000 samples to obtain 95% confidence intervals on model parameters. Points of subjective equality (PSEs) for the two groups were estimated using the fitted sigmoid curves resulting from the logistic regressions to interpolate the relative point levels at which alcohol and snacks were equally likely to be chosen. In addition, we further calculated individual PSEs and characterized each participant as having an overall preference for alcohol, snack or neither of the two (based on 95% bootstrapped CIs of the PSE overlapping 0). Frequencies of light and heavy drinkers in each category were then compared using Chi^2^ test.

Finally, we calculated the association between alcohol use severity and percentage of alcohol choice. First, we performed a lme model with AUDIT, relative point level, and group as independent variables and sex as covariate. Percentage of alcohol choice data were fitted using logistic regression across all participants relative to AUDIT scores, separately for each relative point level. Parametric bootstrapping was performed with 1000 samples to obtain 95% confidence intervals on model parameters. Slopes were obtained from the model fit.

Data were analyzed using R version 4.0.3 with the quickpsy package version 0.1.5.1 (Linares & López-Moliner 2016) and SPSS version 28.0.1. Graphs were created in R with the ggplot2 package version 3.3.6 (Wickham 2016).

## Results

### Participants

Demographic characteristics and questionnaire scores for participants are presented in Table 1. Heavy drinkers had higher AUDIT scores compared to light drinkers. In the heavy drinking group, 100% had AUDIT scores ≥ 7, indicating that all heavy drinkers likely meet criteria for moderate or severe AUD, based on previously presented cutoff scores (Källmén et al. 2019). Conversely, 40% of light drinkers fit the same criteria. The difference in frequency was statistically significant (*χ*^*2*^ = 22.9, *df* = 1, *p* < 0.001). Heavy drinkers scored higher on impulsiveness on the BIS, and scored lower on the NEO-FFI factor Conscientiousness (Table 1). There were no significant differences between the groups on other personality traits, age, risk of drug use disorders as measured with DUDIT, or family history of AUD as measured with the Family tree questionnaire (Table 1). AUDIT scores and self-reported drinks per week were significantly correlated (*r* = 0.74, *p* < 0.001).

**Table 1:** Sample characteristics

	LD *n* = 30	HD *n* = 30	TOTAL *n* = 60	t-test	Effect size
Female, *n*	15	15	30		
Male, *n*	15	15	30		
Age	24.93 ± 6.20, 30	24.03 ± 5.45, 30	24.48 ± 5.81, 60	*p* = 0.55	${\eta }^{2}$= 0.006
Drinks/week	5.1 ± 3.43, 30	16.47 ± 3.74, 30	10.78 ± 6.74, 60	*p* < 0.001	${\eta }^{2}$= 0.722
AUDIT	7.57 ± 3.91, 30	15.17 ± 4.97, 30	11.37 ± 5.86, 60	*p* < 0.001	${\eta }^{2}$= 0.428
DUDIT	1.3 ± 1.78, 30	1.6 ± 2.19, 30	1.45 ± 1.99, 60	*p* = 0.56	${\eta }^{2}$= 0.006
BIS	56.59 ± 7.07, 29	61.63 ± 9.61, 30	59.15 ± 8.76, 59	*p* = 0.03	${\eta }^{2}$= 0.084
* Attention*	7.34 ± 1.59, 29	8.6 ± 2.27, 30	7.98 ± 2.05, 59	*p* = 0.02	${\eta }^{2}$= 0.096
* Cognitive instability*	6.03 ± 1.82, 29	5.9 ± 1.81, 30	5.97 ± 1.80, 59	*p* = 0.78	${\eta }^{2}$= 0.001
* Motor*	14.31 ± 3.22, 29	15.67 ± 3.43, 30	15 ± 3.37, 59	*p* = 0.12	${\eta }^{2}$= 0.041
* Perseverance*	7.31 ± 1.54, 29	7.57 ± 1.61, 30	7.44 ± 1.57, 59	*p* = 0.54	${\eta }^{2}$= 0.007
* Self control*	11.73 ± 3.08, 30	13.57 ± 3.02, 30	12.65 ± 3.17, 60	*p* = 0.02	${\eta }^{2}$= 0.085
* Cognitive complexity*	10.52 ± 3.27, 29	11.27 ± 2.66, 30	10.90 ± 2.98, 59	*p* = 0.34	${\eta }^{2}$= 0.016
FTQ	0.84 ± 0.15, 30	0.03 ± 0.08, 28	0.06 ± 0.12, 58	*p* = 0.11	${\eta }^{2}$= 0.045
NEO-FFI					
* Neuroticism*	45.6 ± 7.14, 30	44 ± 8.28, 30	44.78 ± 7.7, 60	*p* = 0.44	${\eta }^{2}$= 0.011
* Extraversion*	55.5 ± 10.4, 30	59.6 ± 8.51, 30	57.57 ± 9.64, 60	*p* = 0.1	${\eta }^{2}$= 0.045
* Openness*	49.9 ± 10.54, 30	51.3 ± 8.97, 30	50.62 ± 9.73, 60	*p* = 0.57	${\eta }^{2}$= 0.006
* Agreeableness*	55.23 ± 6.4, 30	53.2 ± 8.1, 30	54.22 ± 7.31, 60	*p* = 0.29	${\eta }^{2}$= 0.020
* Conscientiousness*	54.03 ± 8.78, 30	44.67 ± 12.79, 30	49.35 ± 11.86, 60	*p* < 0.005	${\eta }^{2}$= 0.159

Baseline measures for participants who completed the study. *AUDIT* Alcohol Use Disorder Identification Test, scores of > 20 indicate high likelihood of AUD, *DUDIT* Drug Use Disorder Identification Test, *BIS* Barratt Impulsivity Scale, *FTQ* Family Tree Questionnaire, *NEO-FFI* abbreviated Five Factor Inventory personality assessment

### Concurrent choice alcohol-food task results

The lme model on logit-transformed data showed that percentage of alcohol choice was predicted by group (*F*_1,56_ = 8.3, *p* = 0.006, ${\eta p}^{2}$ = 0.13), and relative point level (*F*_2,112_ = 129.8, *p* < 0.001, ${\eta p}^{2}$ = 0.70). No significant group by relative point level interaction was identified (*F*_2,112_=0.10, *p* = 0.90). Percentage of alcohol choice was greater in heavy drinkers (HD = 54.9%, 95% CI = [38.4, 70.4]; LD = 27.2%, 95% CI = [16.8, 41.0]). In addition, percentage of alcohol choice increased as a function of increased relative point to alcohol (relative point level -2 = 4.9, 95% CI = [3.1, 7.7]; relative point level 0 = 37.1, CI = [24.3, 52.1]; relative point level + 2 = 91.0, CI = [84.0, 95.1]). A sex by relative point level interaction was identified (*F*_1,116_ =9.5, *p* = 0.002, ${\eta p}^{2}$= 0.08), with greater percentage of alcohol choice in males compared to females at relative point level + 2 (*t* = -2.95, *p* = 0.043). Sex showed a marginal influence that did not reach statistical significance (*F*_1,56_=3.6, *p* = 0.06, ${\eta p}^{2}$= 0.06), with greater percentage of alcohol choice in males compared to females (female = 31.4, CI = [19.8, 46.1]; male = 49.9, CI = [33.5, 66.3]).

**Fig. 3:**
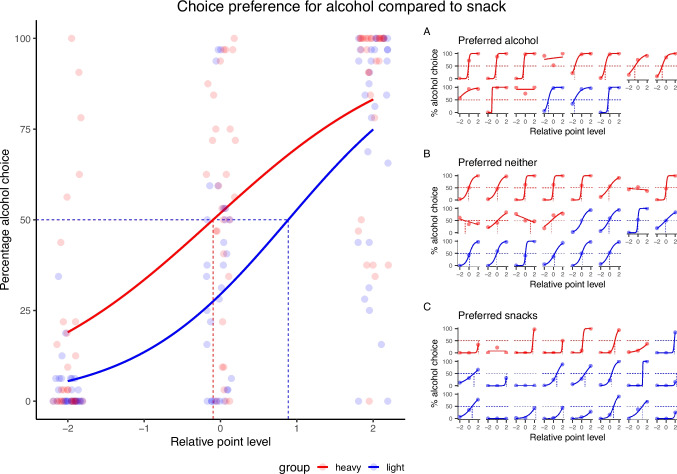
Concurrent Choice Alcohol Food task result. The percentage of trials on which alcohol was chosen (y-axis) is shown according to the relative point level (x-axis: -2, 0 or + 2), for both heavy (red) and light (blue) drinkers. Left. The lme model on logit-transformed data showed that, compared to LD, HD had increased percentage of alcohol choice (*F*_1,56_ = 8.3, *p* = 0.006, ${\eta p}^{2}$ = 0.13). In both groups, increased choice preference for alcohol was observed when relative point level was in favor or alcohol (*F*_2,112_ = 129.8, *p* < 0.001, ${\eta p}^{2}$ = 0.70). For HD, the slope value was 0.76 (95% CI = [0.71, 0.82]), whereas for LD, the slope value was 0.98 (95% CI = [0.91, 1.06]). For HD, the PSE was -0.1 (95% CI = [-0.2, 0.02]). For LD, the PSE was + 0.9 (95% CI = [0.8, 0.9]). Individual data points reflect untransformed data. Right. Logistic regression curves in separate panels for each participant. The percentage of trials on which alcohol was chosen (y-axis) is shown according to the relative point level (x-axis: -2, 0 or + 2). (**A**) 11 of 30 HD and 3 of 30 LD preferred alcohol (**B**) 7 of HD and 17 of LD preferred snacks and (**C**) 12 of HD and 10 of LD had no significant preference

Group-level logistic regression curves are presented in Fig. 3. Light and heavy drinkers had different points of subjective equality (PSEs) for the relative value of alcohol vs. snacks (Fig. 3B). For HD, the PSE was -0.1 (95% CI = [-0.2, 0.02]). For light drinkers, it was + 0.9 (95% CI = [0.8, 0.9]). This indicates that heavy drinkers attributed approximately 1 more unit of value to a choice of alcohol over snack compared to light drinkers. For heavy drinkers, the slope of the regression curve was 0.76 (95% CI = [0.71, 0.82]), whereas for light drinkers, it was 0.98 (95% CI = [0.91, 1.06]). Because the confidence intervals for this metric did not overlap for the two groups, this indicates that, while both groups showed sensitivity to relative choice value, this sensitivity was significantly lower in heavy drinkers.

Individually fitted logistic regression curves are presented in Fig. 3. Because the study was powered to detect group effects, confidence intervals are too large to precisely estimate individual level PSEs, but are sufficient to determine individual overall preferences. Of the 30 heavy drinkers, 11 preferred alcohol, 7 preferred snacks, and 12 neither of the two. Of the light drinkers, 3 preferred alcohol, 17 preferred snacks and 10 neither of the two. Despite some within-group heterogeneity, a significant difference in frequencies was observed *(χ*^*2*^ (2, *N* = 60) = 8.92*, p* = 0.012*)*, driven by between group differences in the preferring alcohol category (Fig. 3A) (*χ*^*2*^ = 6.97, *df* = 1, *p* = 0.008) and in the preferring snack category (Fig. 3C) (*χ*^*2*^ = 5.95, *df* = 1, *p* = 0.015). No significant difference was found in the remaining category (Fig. 3B) (*χ*^*2*^ = 0.29, *df* = 1, *p* = 0.59).

**Fig. 4:**
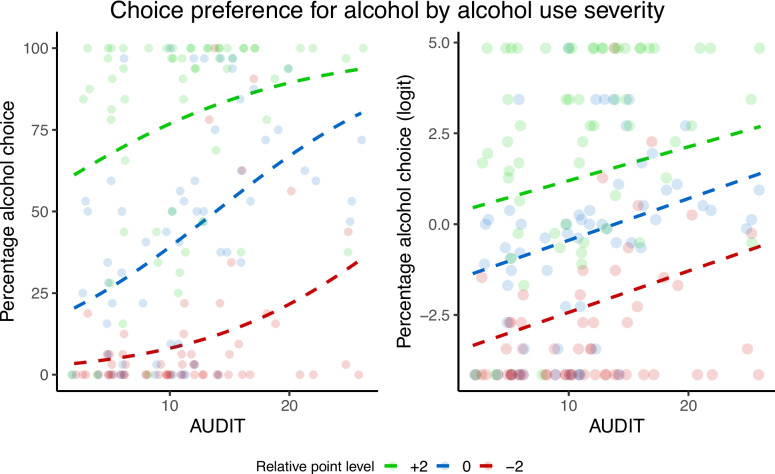
Choice preference for alcohol relative to alcohol severity scores. The percentage of trials on which alcohol was chosen (y-axis) is shown according to participants’ AUDIT scores, separately for the relative point level (+ 2 green; 0 blue; or -2 red). Curves were fitted using logistic regression for binomial data for each relative point level, and parametric bootstrapping was performed with 1000 samples. The slopes of the fitted curves were obtained. The two panels show the untransformed data (Left) and the same data and curves plotted, logit transformed (Right), on which scale the curves appear as lines to facilitate visual comparison of the slopes. For a relative point level of -2, the slope was 0.11 (95% CI = [0.09, 0.14]); for a relative point level of 0, the slope was 0.12 (95% CI = [0.10, 0.13]), and for a relative point level of + 2, the slope was 0.09 (95% CI = [0.07, 0.12], logit %)

A positive association between alcohol use severity as measured with the AUDIT, and percentage of alcohol choice was found (Fig. 4). The lme model on logit-transformed data showed that percentage of alcohol choice was predicted by AUDIT (*F*_1,56_ = 12.5, *p* < 0.001, ${\eta p}^{2}$= 0.18), and relative point level (*F*_1,116_ = 43.2, *p* < 0.001, ${\eta p}^{2}$ = 0.27). No significant AUDIT by relative point level interaction was found (*F*_1,116_=0.4, *p* = 0.5). Similarly to the categorical model, a relative point level by sex interaction was found at trend level (*F*_1,116_ =3.7, *p* = 0.06, ${\eta p}^{2}$ = 0.03). No other significant effects were found. The slope of the association between AUDIT scores and percentage of alcohol choice was significant for all relative point levels. For a relative alcohol level of -2, the slope was 0.11 (*p* = 0.02, 95% CI = [0.09, 0.14]), for a level of 0, the slope was 0.12 (*p* = 0.001, 95% CI = [0.10, 0.13]), and for a level of + 2, the slope was 0.09 (*p* = 0.014, 95% CI = [0.07, 0.12]). Overall, these findings indicate that preference for alcohol was associated with alcohol use severity, across all relative point levels.

## Discussion

We used a concurrent, mutually exclusive choice paradigm, and found that choice preference for alcohol-related stimuli is related to both alcohol use level, and to the relative point level of the reward. For both light and heavy drinkers, choice preference for alcohol was sensitive to parametric variation in relative point magnitude associated with the respective rewards. However, heavy drinkers generally chose alcohol over snacks more than light drinkers, and valued alcohol higher than snacks compared to light drinkers. Heavy drinkers evaluated alcohol and snacks equally when the two rewards had approximately the same point value, whereas light drinkers needed alcohol to be associated with approximately one extra point to consider it equal in value to a snack.

Our findings replicate and extend prior findings (Hogarth & Hardy 2018), and are consistent with the proposed conceptualization of mechanisms that promote excessive alcohol seeking and taking in individuals with AUD (Hogarth al. 2019). We replicate the significant association between alcohol use severity and preference for alcohol across all relative point levels (Hogarth & Hardy 2018). We also show that, while alcohol drinking severity affected choice behavior, so did the value of the alternative. In both groups, we observed increased preference for alcohol when alcohol was associated with more points. Thus, in heavy drinkers the ability for value-based decision making, while biased in favor of alcohol, remained sensitive to outcomes. This finding, and the largely parallel left shift of the relationship between relative point value and choice preference supports the hypothesis that the major behavioral feature associated with alcohol use severity is not insensitivity to costs associated with alcohol-seeking behavior (Hogarth 2020; Hogarth & Hardy 2018). By tailoring the alcohol and snack pictures to subjective preference, we substantially improved on the sensitivity of the task, as we observed a larger effect size for relative point difference between heavy and light drinkers than in the original study [(Hogarth & Hardy 2018), ${\eta p}^{2}$ = 0.70 for the first lme using group as predictor and ${\eta p}^{2}$ = 0.27 for the second lme using AUDIT as predictor vs ${\eta p}^{2}$ = 0.14]. This suggests a strengthened relationship between alcohol use characteristics “in the wild” and alcohol choice in the lab.

The findings of our study could be interpreted as evidence that the relative reinforcing efficacy of alcohol is generally increased in heavy compared to light drinkers, reflecting a heightened decision utility of the drug (Berridge & Aldridge 2008). However, behavioral economic analysis suggests that a unitary construct of relative reinforcing efficacy is insufficient to capture motivational properties of rewards, and that a demand curve analysis produces metrics more consistent across single reinforcer and choice approaches (Bickel et al. 2000). Our paradigm was not designed to allow a formal demand curve analysis, but captures metrics likely to reflect two distinct aspects of demand characteristics. First, the largely parallel left-shift of the choice preference curve in heavy drinkers is consistent with a heightened alcohol demand intensity in this group across cost levels. Second, the somewhat lower slope of the preference curve in heavy drinkers is consistent with a modestly attenuated price elasticity of alcohol demand in this group, i.e. a lower change in demand as a function of change in cost.

Based on an analysis of the relationship between the price of alcohol in American cities with self-reported consumption, it has been suggested that price elasticity of alcohol is lower in heavy compared to moderate drinkers (Manning et al. 1995), i.e. that heavy drinkers change their alcohol use less for any given change in price than do moderate drinkers. A subsequent meta-analysis also found that, while there is a highly significant relationship between the cost of alcohol and its consumption in general, the magnitude of this effect is smaller for heavy drinkers than for the general population (Wagenaar et al. 2009). However, a recent meta-analysis of demand characteristics for alcohol determined under experimental conditions in the Alcohol Purchase Task concluded that the effect size for the association between demand intensity and drinking measures is large, while that for price elasticity and drinking measures is low – moderate (Martínez-Loredo et al. 2021). Our finding is indicative of an increase in demand intensity, combined with a modest decrease in price elasticity in heavy drinkers are consistent with findings presented above. Overall, our results are in line with the hypothesis that alcohol seeking in individuals with heavy drinking and AUD primarily reflects changes in value-based choice behavior rather than insensitivity to cost (Hogarth 2020).

This study has both strengths and limitations. By tailoring task stimuli to individual participant preferences, we accounted for subjective preference and also increased ecological validity compared to the original version of the task (Hogarth & Hardy 2018). However, our experimental setup did not replicate a context in which participants would typically engage in drinking alcohol. Nevertheless, despite the artificial setting, we could identify robust between-group differences. It can be speculated that these effects may be even larger in a naturalistic setting. In addition, the robust between-group effects indicate that the implemented task simplifications did not affect the original results (Hogarth & Hardy 2018), making this task suitable for fMRI investigations. Finally, the CCAF task allowed us to identify the behavioral profiles of two populations at different level of alcohol use severity. Based on AUDIT cutoff scores (Källmén et al. 2019), but see also (Ingesson-Hammarberg et al. 2024), all participants in the heavy drinking group met criteria for moderate or severe AUD, with a majority meeting criteria for severe AUD. This indicates that despite the absence of a formal AUD diagnosis, most of heavy drinkers likely experience clinically levels of alcohol use severity. Thus, this task could potentially be used as tool in early-stage evaluation of candidate therapeutics for AUD.
